# Evaluating West Virginia’s Emergency Medicine Workforce: A Longitudinal Observational Study

**DOI:** 10.7759/cureus.13639

**Published:** 2021-03-01

**Authors:** Joseph Hansroth, Scott W Findley, Kimberly D Quedado, Thomas Marshall, Andrew Vucelik, Christopher S Goode

**Affiliations:** 1 Emergency Medicine, West Virginia University School of Medicine, Morgantown, USA

**Keywords:** emergency medicine physician, rural medicine, medical workforce

## Abstract

Objective

Although the urban emergency workforce is well studied, rural departments are less understood. This study seeks to further define the landscape of rural healthcare and expand on previous studies of the West Virginia (WV) workforce.

Methods

During the second quarter of 2019, surveys were sent via email to medical directors’ professional IDs as anonymous survey links. Hard copies were also sent to directors at their hospital addresses. Responses were aggregated with hospitals stratified based on annual census and rural classification. Data was interpreted through descriptive analysis.

Results

Surveys were sent to 53 departments with a 55% response rate. Of the responding hospitals, 15 of 29 were identified as rural. The average state-wide annual hospital census was 29,500 visits with board-certified emergency medicine (EM)-trained physicians covering 67% of shifts. Rural departments have a smaller census and less specialized coverage. Full-time physicians are found to have the strongest ties to WV, with 65% attending medical school, residency, or growing up in the state.

Conclusion

Board-certified EM-trained physicians provide some level of coverage in most emergency departments in WV but remain underrepresented in rural locations. This specialized coverage has increased by 20% in the last 15 years. Additionally, a majority of hospitals have access to basic consulting services (surgery and primary care); however, other specialists are rare in rural WV.

## Introduction

Emergency medicine (EM), compared to most specialties, is still a young and burgeoning field in the house of medicine. When the first emergency rooms opened in the 1950s, physicians from all backgrounds treated patients [1]. Specialty trained physicians began to cover emergency departments (EDs) in the 1970s and the specialty has grown steadily since then [2].Today’s wide variation of practice environments ranges from the large, urban, and academic to small, rural and frontier facilities. Although urban and suburban EDs are most commonly staffed by specialty trained and board-certified physicians, smaller and more rural departments are frequently covered by non-EM board-certified providers such as family medicine physicians, pediatricians, internists, and others [3,4].

Importance and goal of investigation

Comprehensive studies have recently been done nationally on the EM workforce but work needs to be done to specifically characterize rural EM [5]. With visits in rural EDs rapidly increasing, questions remain regarding how to measure acuity levels, annual volumes, assess training, and determine optimal specialty staffing in rural EDs [6-9]. West Virginia (WV), which lies entirely within Appalachia, is well positioned to help define rural healthcare. Although six of the country’s largest cities can be reached within five hours of driving, the population of WV’s largest city is only 46,536 [10]. EDs in WV serve a population of 1.8 million residents, with most residents living in rural areas [11]. The state’s workforce has been studied twice in the past 20 years to evaluate the training and background of the physicians, physician assistants (PAs), and nurse practitioners (NPs) who care for patients [12,13]. An updated study can provide indicators of the level of transformation of the EM care provided in WV.

This study aims to provide updated information on the state of West Virginia’s EM provider workforce and further define the largely unknown clinical coverage of the rural United States. Specifically, the survey evaluated departmental staffing and facilities, physician coverage, training, ties to WV, and consultant coverage.

## Materials and methods

Design and settings

This is a cross-sectional survey-based study. The study has been reviewed and approved by the West Virginia University Institutional Review Board. The online survey was designed and distributed using Qualtrics survey software,Version [May 2019] (Qualtrics, Provo, UT) based on prior WV Workforce Surveys and updated for new metrics.

Participant selection

The survey was distributed via email to all medical directors within the West Virginia American College of Emergency Physicians (WV ACEP) listserv. Surveys were distributed electronically three times between May and September 2019. Emails contained a cover letter and a secure link to the survey. Hard copies were also available with addressed return envelopes and fax numbers as an alternative to electronic submission. Follow-up phone calls were made in September 2019 to facilities to discuss study completion and to assist with any questions medical directors may have had.

Data collection

The data collected focused on several descriptive areas of each department. Department size, annual census, available specialty services, telehealth services, use of advanced practice providers (APPs), and trauma designation all aimed to provide a comprehensive evaluation of resources available to providers while working in each department. Staffing information was also collected with the focus on full-time versus part-time and third-party company hired, locum physician utilization. We also evaluated each department for personal and professional ties to WV.

Analysis

Survey data was either collected electronically or through manual input by the authors. Data was exported from Qualtrics and analysed in Microsoft Excel (Mac 2011 version 14.4.8), and results were compared to the two prior WV Workforce Surveys for temporal relationships and trends. The value cutoffs for the small (S), medium (M) and large (L) facilities were derived through consensus opinion from community EM practitioners within our department. Hospitals with less than 20,000 yearly visits (S) were hypothesized to trend toward single-coverage facilities, have limited subspecialty availability, be more dependent on transfer and more likely to be Critical Access Hospitals (CAH). Volumes of 20,000-40,000 were hypothesized to represent typical community hospitals with ED provider overlap, more access to resources and may be less dependent on transfer networks. Volumes over 40,000 yearly ED visits, grouped as large, were thought to represent receiving hospitals with comprehensive or nearly comprehensive care. Many rural facilities are federally designated CAH and have an arbitrary bed cap of 25 inpatient beds [14]. We sought to maintain anonymity of the respondents, and number of acute care beds is widely available. We believe ED volumes better correlate with ED use and chose to group hospitals into three categories for the purpose of this analysis.

In a second stratification, analysis of rural departments was completed using the same definition as the 2004 West Virginia Workforce study (county population <30,000 or the only hospital in a county), and was performed as a retrospective analysis using the most recent US Census data, trauma center data, and cross-referencing study responses [13]. This was possible based on the state’s unique geography and distribution of hospitals.

## Results

Twenty-nine completed surveys were collected during the study period. Electronic responses accounted for 97% data with the final response being collected via returned hard copy response.

**Figure 1:**
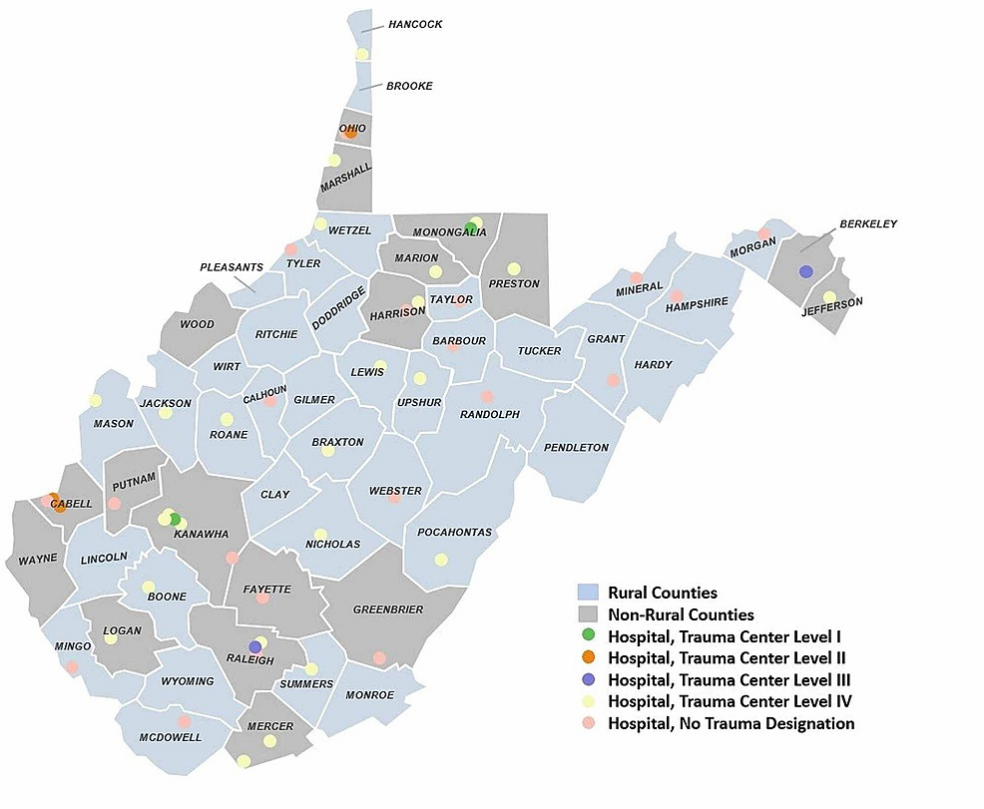
County Population Stratification and Hospital Trauma Designation Map Courtesy: This figure was created by Tracy Sun, MPH, West Virginia Clinical & Translational Science Institute, and is critical to the geographic visualization of hospitals in West Virginia.

From the responses received (Table 1), the average West Virginia Emergency Department was 20 beds (range 4-48 beds) with an annual census of 29,500 (range 6,000-60,000). Fifteen of 29 (52%) of facilities were identified as rural using the 2004 criteria [13]. Of the cohort studied, no large facilities (0/9), 71% (5/7) medium facilities, and 77% (10/13) small facilities met criteria for rural designation. Dispositions were similar between small, medium, and large facilities, as well as rural and non-rural, as highlighted in Table 1. The results indicate large hospitals and non-rural hospitals housed larger numbers of beds with an average of 29 beds compared with 11 beds at the rural facilities. Trauma designations were grouped as Levels 1 to 3 as defined by the American College of Surgeons criteria and Level 4 being a state designation for facilities to guide trauma team activation and specialty surgeon coverage (Figure 1) [15]. For responding hospitals' designation, results are as follows: two of two Level 1 hospitals (100% response rate), two of three Level 2 hospitals (67%), two of three Level 3 hospitals (67%), 17 of 25 Level 4 hospitals (68%), and six of 20 with no trauma designation (30%). Telemedicine services have been adopted across the state as well, with 59% of respondent hospitals. Rural hospitals report using telemedicine slightly more than non-rural hospitals with 60% and 57% utilization, respectively.

**Table 1:** Emergency Department Demographics

Responding Hospital’s Stratification	Total		Rural	Non-Rural		Small (<20k)	Medium (20-40k)	Large (>40k)
Respondents	29		15	14		13	7	9
Total visits	827,055		231,000	596,055		145,855	194,000	487,200
Average visits	29,500		15,400	42,575		11,200	27,700	54,100
Number of beds	20		11	29		8	19	38
Percent discharge rate	74%		70%	78%		77%	76%	69%
Level 1	2		0	2		0	0	2
Level 2	2		0	2		0	0	2
Level 3	2		0	2		0	0	2
Level 4	17		10	7		8	6	3
No trauma	6		5	1		5	1	0
Telemedicine available	59%		60%	57%		33%	100%	54%

ED provider staffing characteristics

Staffing of West Virginia EDs was a key feature of the study and can be used as a comparison of the 2004 workforce in the state [13]. Reported average ED shift coverage translated to an average of 77% of shifts covered by full-time physicians across all respondents (Table 2); part-time physicians and locum physicians cover the remaining 23% of shifts. Locum physicians are utilized in staffing in 48% of responding hospitals.

**Table 2:** Provider Staffing of West Virginia Emergency Departments BC/BE, board-certified/board eligible; APP, advanced practice provider

All Physicians	Total		Rural	Non-Rural		Small (<20k)	Medium (20-40k)	Large (>40k)
Total physicians	385		138 (36%)	247 (64%)		99 (26%)	107 (28%)	179 (46%)
Physicians per department	13		9	18		8	15	20
Reported percentage of shift coverage								
Full-time coverage	77%		73%	81%		76%	76%	79%
Part-time coverage	15%		19%	13%		19%	9%	15%
Locums coverage	6%		8%	6%		5%	7%	8%
BC/BE physician breakdown								
Percentage of BC/BE physicians	243 (63%)		61 (44%)	183 (74%)		23 (23%)	93 87%	156 87%
BC/BE full-time EM	273 (71%)		51 (37%)	178 (72%)		17 (17%)	88 (82%)	154 (86%)
BC/BE part-time EM	227 (59%)		61 (44%)	188 (76%)		24 (24%)	92 (86%)	161 (90%)
% Shifts covered BC/BE	67%		53%	76%		23%	85%	93%
APP breakdown								
Departments reporting APPs	79%		60%	100%		69%	71%	100%

EM board-certified/board eligible (BC/BE) physicians account for 67% of the total shifts worked; the most common location for BC/BE physicians to work is in a large non-rural facility. There remains a variation in the specialty-trained physicians as the small group of responding hospitals only has a 23% rate of EM-trained physician coverage, whereas in the medium and large hospital cohorts, BC/BE physicians cover 85% and 93% of shifts, respectively. Rural analysis shows EM-trained physicians cover 42% of rural designated hospital shifts and 74% of shifts in the non-rural group.

APPs, defined as physician assistants and nurse practitioners, provide a meaningful extension of care for emergency departments across the state. APPs are utilized for some manner of patient care in 79% of responding departments. APPs are utilized in all non-rural hospitals and in 60% of rural hospitals. In departments utilizing APPs, it is estimated 21% of patients are evaluated primarily by PAs and 11% are evaluated primarily by NPs. The main emergency department is where 61% of APPs work; 35% work in the lower acuity areas of the ED.

Ties to West Virginia are likely linked to staying within the state as a physician, with 57% of the physicians included in this survey growing up in West Virginia, 57% attending medical school in the state, and 58% completing residency in West Virginia (Table 3). Full-time physicians were much more likely to have in-state ties than part-time physicians or locum physicians, as noted in Table 3.

**Table 3:** Physician Ties to West Virginia

	Grew Up (In-State)	Medical School (In-State)	Residency (In-State)
All physician groups	57%	57%	58%
Full time	65%	67%	62%
Part time	38%	39%	44%
Locums	18%	17%	18%

Full-time consultant services were also evaluated in the survey (Table 4). The most common consultants available, in descending order, were general surgery, internal medicine, orthopaedics, and family medicine. Ophthalmology, cardiovascular surgery, hand surgery, plastic surgery, vascular surgery, ENT, neurosurgery, and psychiatry were the services least available, all of which were available to less than 40% of emergency departments in the survey and none of these services were available in small facilities. Rural hospitals, specifically, have fewer consultant services available. Although most rural hospitals have general surgery, internal medicine, family medicine, and orthopaedics, coverage for pediatrics, OB/GYN, and cardiology are less common. No other specialty is available at more than two of 15 responding rural facilities.

**Table 4:** Consulting Services at Hospitals

Consulting Service	Total		Rural	Non-Rural		Small (<20k)	Medium (20-40k)	Large (>40k)
General surgery	86.2%		73.3%	100%		69.2%	100.0%	100.0%
Internal medicine	79.3%		60%	100%		69.2%	71.4%	100.0%
Orthopaedics	72.4%		53.3%	92.9%		46.2%	85.7%	100.0%
Family medicine	69.0%		60%	78.6%		76.9%	42.9%	77.8%
Pediatrics	62.1%		40%	85.7%		23.1%	85.7%	100.0%
OB/GYN	58.6%		40%	78.6%		7.7%	100.0%	100.0%
Cardiology	51.7%		20%	85.7%		7.7%	71.4%	100.0%
Pulmonology	48.3%		13.3%	85.7%		7.7%	57.1%	100.0%
Nephrology	44.8%		13.3%	78.6%		7.7%	42.9%	100.0%
Urology	44.8%		6.7%	85.7%		15.4%	28.6%	100.0%
Neurology	41.4%		6.7%	78.6%		7.7%	28.6%	100.0%
Gastroenterology	41.4%		13.3%	71.4%		7.7%	42.9%	88.9%
Psychiatry	34.5%		13.3%	57.1%		0.0%	42.9%	77.8%
Neurosurgery	34.5%		0%	71.4%		0.0%	14.3%	100.0%
ENT	34.5%		6.7%	64.3%		0.0%	28.6%	88.9%
Vascular	31.0%		0%	64.3%		0.0%	14.3%	88.9%
Plastics	20.7%		0%	42.9%		0.0%	14.3%	55.6%
Hand	17.2%		0%	35.7%		0.0%	14.3%	44.4%
CT surgery	17.2%		0%	35.7%		0.0%	14.3%	44.4%
Ophthalmology	13.8%		0%	28.6%		0.0%	14.3%	33.3%

Focusing on the comparison with the 2004 data, the 2019 survey uncovered gains in total number of physicians with the full-time status remaining consistent (Table 5) [13]. Specialty training in EM has increased by 20% since prior research. With regard to ties to WV, data demonstrated an increase in ties to WV for physicians remaining in the state across all areas. Despite a stable population on the US Census, ED visits have increased by 20% over the same timeframe as well.

**Table 5:** Comparative Data for 2004 and 2019 EM, emergency medicine; WV, West Virginia; BC/BE, board-certified/board eligible

	2004	2019
Responses	39 (71%)	29 (55%)
Mean census	23,375 (2,000-60,000)	29,500 (6,000-60,000)
Total physicians	310	385
Full-time employment	76%	77%
Full-time EM BC/BE	51%	67%
Grew up in WV	47%	57%
WV med school	53%	57%
WV residency	43%	58%

## Discussion

This study reviews key aspects of EM care and departmental characteristics in West Virginia during 2019. Results build both on the recent national EM workforce study and on prior research within this state. Characteristics of providers in West Virginia show some stable features along with some developing trends. Full-time physicians with personal or professional ties to the state cover most shifts, and the number of EM board-certified/board eligible physicians covering shifts has increased by 20% since the last state workforce evaluation in 2004 [13]. APPs have increased their presence in the state’s emergency departments since 2004 with an overall increase of 54% from the 2004 staffing numbers. This is consistent with the overall national growth in their respective fields; for example, the National Commission on Certification of Physician Assistants (NCCPA) reports a 53.8% increase in positions since 2010 and the Bureau of Labor Statistics is reporting substantial growth potential for both PAs and NPs over the next 10 years [16,17]. The departments in which providers work are widely varied as well, from academic Level 1 trauma centers to rural departments with minimal supporting services. While most hospitals (97%) have available consultants, only 41% of hospitals have neurology and 35% have psychiatry available and both of these specialties are concentrated at larger hospitals. In descending order, the most common specialties available from EDs are general surgery, internal medicine, family medicine, orthopaedics, and urology. Of note, OB/GYN services are available at only 36% of hospitals and are downtrending based on a number of smaller delivery wards closing across the state due to high operating costs and low census [18].

Bennett et al. recently published a national EM workforce analysis that showed that only 8.5% of EM providers nationwide practice in a rural setting based on US Department of Agriculture Urban Influence Codes [5]. The South Atlantic region, which includes West Virginia, has 5.6% of EM providers working in rural departments. Our study shows that 36% of the West Virginia EM workforce works in a rural facility using the definition of rural as stratified based upon county population.

The trend in increasing BC/BE EM coverage is multifactorial, likely resulting from an increase in medical school positions since 2004, an overall increased number of EM residency positions, and successful recruitment and retention of students and residents. Since the 2004 study, there have been several changes to emergency medicine within the state. Three hospitals have closed, including one academic center with an EM residency in 2019. Despite this closure, residency complement changes have resulted in a net increase of four graduating EM residents per year.

The total number of providers in rural EDs has increased substantially since 2004, with an overall increase of 44%. However, the increase of BC/BE EM coverage has been seen primarily at larger hospitals with smaller and rural hospitals showing more modest gains in EM coverage. We expect the trend toward increased BC/BE EM provider coverage to continue as the large academic system in the state has integrated multiple small community hospitals and has been successful in recruiting BC/BE physicians to staff those facilities. In addition to flexible staffing contracts, a rural EM experience for senior EM residents has been created to increase exposure.

Looking toward the future of EM care in West Virginia shows several areas of promise highlighted by results of this study. Although a majority of patients are discharged home after an ED visit, nearly 10% (9.26%, range 0 to 20%) are transferred to a higher level of care [16]. The hospitals with the highest transfer rates are those with smaller census and fewest consultants. The clinical outcomes of transferred patients is an area for future research - evaluating transfer times in relation to definitive care and possible increased mortality in comparison to patients who present initially to a larger hospital. Telemedicine is a growing option for rural EDs across the state with 59% already using either neurology or psychiatry services. This has come in response to an overall lack of specialty availability. Once these programs are well established, tele-EM, tele-ICU, and other specialties can be introduced or expanded utilizing the same technology.

Limitations

There are several limitations to this study, including a modest response rate of 55% (29/53) with results skewed for larger facilities due to higher response rates. Because of the reduced response, limited direct comparisons can be made with the 2004 study. The responding hospitals report cumulative annual visits of 827,000; Kaiser Family Foundation data estimates the West Virginia state ED visits to be 683 visits per 1,000 population [19]. Through extrapolation, it is estimated to be 1.2 million ED visits based on a WV census population of 1.8 million. Given our sample size estimates, 69% of visits with a 55% response rate, most facilities that did not respond are small departments with no trauma center designation. Since survey results were anonymously submitted, specific tracking of which facilities responded was impossible. Blinding was used in order to encourage accurate responses from medical directors. Some physician data may include providers who are counted multiple times as it is common for providers to work at several hospitals. Data on patient disposition, physician and APP coverage, and ties to West Virginia was collected using a sliding scale with a set interval of 10% requiring medical directors to provide rounded estimates. The survey did not include a way for medical directors to identify overlap between physician ties to West Virginia. The definition of consulting services may have been interpreted differently by medical directors, yielding an incomplete picture of what services are available at some hospitals.

## Conclusions

This medical director survey of the West Virginia EM workforce highlights several aspects of healthcare and several areas of improvement from prior research. Despite a static and aging population, ED visits have risen by 20%. The number of BC/BE EM physicians within the state has increased by 20% in the last 15 years. APP utilization mirrors the national trend with a 28% increase as well. Access to advanced diagnosis and intervention for cardiovascular and neurological disease is still a challenge in small hospitals. Telemedicine may offer a solution, providing specialist care to rural hospitals that otherwise have very limited consulting resources. Education continues to expand via new and increased residency programs to rural EM rotations now being provided with the goal of increasing the penetration of EM-trained specialists in state’s communities.
